# Mr. Keyser's Proposal

**Published:** 1921-06-11

**Authors:** F. J. Frankau

**Affiliations:** Deputy Treasurer. St. George's Hospital, S.W. 1


*To the Editor of* The Hospital.

### Mr. Keyser's Proposal.

*To the Editor of* The Hospital.

Sir,—With reference to your request in your article under this heading last week, in my view, whilst this scheme may be successful in the country, where there is usually only one hospital available, it is not likely to be so in the Metropolis. A subscriber of even 2d. a week will consider that for this he is entitled (1) to immediate attention at (2) the hospital of his choice, and anyone with knowledge of our London hospitals won't realise that this is not feasible. The subscriber will never understand what ailments are suitable for hospital treatment, or that there are such things as waiting-lists. Furthermore, most hospitals have at present no legal power to enter into *contracts* for the treatment of the sick, still less for the treatment of those who are well at the time of contracting. This system would, I fear, lead to endless friction between the subscribers, practitioners, and the hospitals. The organisation involved, if there is to be a "clearing" of members amongst the hospitals, would be a great drain on the funds, and the patients (as I said above) would not like being allotted to any other hospital than the one of their choice.—Yours faithfully,

F. J. Frankau, Deputy Treasurer.

St. George's Hospital, S.W. 1,
June 7, 1921.

